# Identification and immune characteristics of molecular subtypes related to fatty acid metabolism in idiopathic pulmonary fibrosis

**DOI:** 10.3389/fnut.2022.992331

**Published:** 2022-09-23

**Authors:** Fan Yang, Zhaotian Ma, Wanyang Li, Jingwei Kong, Yuhan Zong, Bilige Wendusu, Qinglu Wu, Yao Li, Guangda Dong, Xiaoshan Zhao, Ji Wang

**Affiliations:** ^1^College of Traditional Chinese Medicine, Beijing University of Chinese Medicine, Beijing, China; ^2^National Institute of TCM Constitution and Preventive Medicine, Beijing University of Chinese Medicine, Beijing, China; ^3^Institute of Ethnic Medicine, Beijing University of Traditional Chinese Medicine, Beijing, China; ^4^Department of Clinical Nutrition, Chinese Academy of Medical Sciences – Peking Union Medical College, Peking Union Medical College Hospital (Dongdan Campus), Beijing, China; ^5^College of Traditional Chinese Medicine, Shandong University of Traditional Chinese Medicine, Jinan, China; ^6^School of Chinese Medicine, Southern Medical University, Guangzhou, China

**Keywords:** fatty acid metabolism-related genes, idiopathic pulmonary fibrosis, diagnostic model, NK cell, molecular subtype

## Abstract

**Background:**

Although fatty acid metabolism has been confirmed to be involved in the pathological process of idiopathic pulmonary fibrosis (IPF), systematic analyses on the immune process mediated by fatty acid metabolism-related genes (FAMRGs) in IPF remain lacking.

**Methods:**

The gene expression data of 315 patients with IPF were obtained from Gene Expression Omnibus database and were divided into the training and verification sets. The core FAMRGs of the training set were identified through weighted gene co-expression network analysis. Then, the fatty acid metabolism-related subtypes in IPF were identified on the basis of k-means unsupervised clustering. The scores of fatty acid metabolism and the expression of the fibrosis biomarkers in different subtypes were compared, and functional enrichment analysis was carried out on the differentially expressed genes between subtypes. A random forest model was used to select important FAMRGs as diagnostic markers for distinguishing between subtypes, and a line chart model was constructed and verified by using other datasets and rat models with different degrees of pulmonary fibrosis. The difference in immune cell infiltration among subtypes was evaluated with CIBERSORT, and the correlation between core diagnostic markers and immune cells were analyzed.

**Results:**

Twenty-four core FAMRGs were differentially expressed between the training set and normal samples, and IPF was divided into two subtypes. Significant differences were observed between the two subtypes in biological processes, such as linoleic acid metabolism, cilium movement, and natural killer (NK) cell activation. The subtype with high fatty acid metabolism had more severe pulmonary fibrosis than the other subtype. A reliable construction line chart model based on six diagnostic markers was constructed, and *ABCA3* and *CYP24A1* were identified as core diagnostic markers. Significant differences in immune cell infiltration were found between the two subtypes, and *ABCA3* and *CYP24A1* were closely related to NK cells.

**Conclusion:**

Fatty acid metabolism and the immune process that it mediates play an important role in the occurrence and development of IPF. The analysis of the role of FAMRGs in IPF may provide a new potential therapeutic target for IPF.

## Introduction

Idiopathic pulmonary fibrosis (IPF) is an interstitial lung disease that is characterized by dry cough, dyspnea, and progressive lung function decline (1). The pathological features of IPF include persistent damage to alveolar epithelial cells, myofibroblast differentiation, and extracellular collagen deposition (2). The average survival of IPF is only 3–5 years (3). IPF has highly heterogeneous clinical manifestations, and its disease course is highly unpredictable essentially because its exact pathogenesis has not yet been fully elucidated (4, 5). Therefore, considering that existing treatment options can only decelerate the progression of IPF without preventing or reversing it, exploring the causes of IPF heterogeneity on the basis of molecular typing and identifying corresponding biomarkers is an effective strategy for meeting the goal of precision medicine.

Studies have shown that the number and types of components involved in fatty acid metabolism in patients with IPF are disordered. The level of serum total fatty acids in patients with IPF is significantly higher than that in the normal population (6). However, the level of stearic acid in the lung tissue of patients with IPF is lower than that in the lung tissue of normal subjects. Stearic acid supplementation can inhibit the *TGF-*β*/SMAD2/3* signal transduction pathway, which is the main signal transduction mechanism in the progression of pulmonary fibrosis, and can induce the expression of other profibrotic mediators to promote a series of processes, including collagen deposition and extracellular matrix remodeling, synergistically (7, 8). The profibrotic effect of TGF-β is also related to fatty acid metabolism. Fatty acid synthase (*FASN*) is an essential anabolic enzyme that is responsible for the *de novo* synthesis of fatty acids. The level of *FASN* is directly related to the degree of pulmonary fibrosis in a mouse model of pulmonary fibrosis. After the inhibition of *FASN* expression, the degree of TGF-β-induced lung fibrosis is significantly reduced (9). Moreover, the immune cells and immune responses in IPF are closely related to fatty acid metabolism. Macrophages play an important role in the pathogenesis of IPF. Macrophages form through the phagocytosis of extracellular oxidized phospholipids and produce additional TGF-β, which aggravates pulmonary fibrosis (10). Clinical studies have also noted a strong link between fatty acid metabolism and IPF. Statins are traditional drugs for regulating lipid metabolism. A recent randomized, double-blind, placebo-controlled clinical trial suggested that statins can reduce the number of hospitalizations and mortality in patients with the acute exacerbation of IPF (11). Nevertheless, existing studies have only revealed the relationship of fatty acid metabolism and fatty acid metabolism-related genes (FAMRGs) with IPF, and a comprehensive analysis of the role of FAMRGs in the occurrence and development of IPF combined with the immune microenvironment of lung tissue remains lacking.

In this study, we hypothesized that FAMRGs are closely related to the immune microenvironment of lung tissue in IPF. We screened out the key FAMRGs associated with IPF and compared the differences in immune cell infiltration under different FAMRG expression patterns. A diagnostic model for predicting high-risk FAMRG subtypes was established on the basis of machine learning methods, and the core genes constituting the model were validated in other datasets and animal experiments. The results of our study provide references for the application of fatty acid metabolism as a therapeutic target for IPF.

## Materials and methods

### Data source

The IPF-related microarray datasets GSE32537, GSE53845, GSE10667, GSE110147, and GSE150910 were downloaded from the Gene Expression Omnibus (GEO) database (https://www.ncbi.nlm.nih.gov/geo/). These datasets included lung tissue samples from 315 IPF and 187 normal individuals, among which GSE53845, GSE10667, GSE110147, and GSE150910 were used as external verification sets (12–15). Raw data were batch-corrected to remove batch effects for further analysis. Differentially expressed genes (DEGs) were screened by using the “*limma*” package with *P* < 0.05 and |log2FC| > 0.585 indicating statistically significant differences (16). The FAMRGs were obtained from the hallmark gene sets in the Molecular Signature Database (https://www.gsea-msigdb.org/gsea/msigdb/). A total of 1428 genes were obtained for further analysis.

### Weighted gene co-expression network analysis

The fatty acid metabolism gene set enrichment score (FMS) of each sample was calculated through gene set variation analysis (GSVA), and weighted gene co-expression network analysis was performed by using the R package “*WGCNA*” in accordance with the score (17, 18). The adjacency matrix consisted of weighted correlation coefficients, which transformed the adjacency matrix into a topological overlap matrix and a corresponding dissimilarity matrix. Then, hierarchical clustering was carried out to identify modules, construct a systematic clustering diagram, and divide similar gene expression profiles into different modules. Finally, Pearson correlation analysis was used to evaluate the correlation among three phenotypes (FMS, normal control group, and IPF group) and the genes contained in each module.

### Classification and functional enrichment analysis of FAMRG-related subtypes in IPF

Unsupervised cluster analysis was performed on patients with IPF to identify different subtypes on the basis of the key FAMRGs in the related module genes obtained through WGCNA. Meanwhile, principal component analysis (PCA) was used to calculate the fatty acid metabolism level of each sample to obtain the fatty acid metabolism score. A consensus clustering algorithm was used to evaluate the cluster numbers and robustness. The R package “*ConsensusClusterPlus*” implemented the above steps for 1000 iterations to guarantee the robustness of classification (19). Gene set enrichment analysis (GSEA) was performed on the gene expression matrix by applying the “*clusterProfiler*” package, and “*c2.cp.kegg.v7.0.symbols.gmt*” was selected as the reference gene set. The “*ggplot2*”, “*pathview*”, and” “*circlize*” packages were used to perform Gene Ontology (GO) and Kyoto Encyclopedia of Genes and Genomes (KEGG) enrichment analyses on DEGs (20–22).

### Screening, validation, and immunomicroenvironment analysis of diagnostic markers in the different fatty acid metabolism subtypes of IPF

Random forest (RF), a machine learning method that is widely used in the research on diagnostic models, can accurately calculate the importance of each feature in the dataset (23). In this study, the “*randomForest*” package was utilized to select the key differential genes between subtypes as diagnostic markers, and the “*rms*” package was used to construct the prognostic programs (24). Then, a calibration curve was applied to evaluate the predictive ability of the nomogram model, and the clinical value of the constructed model was evaluated by utilizing decision curve analysis (DCA) and a clinical impact curve (25). The key differential genes obtained through screening were verified in the validation set, and the genes with superior diagnostic efficiency were identified as diagnostic markers. The degree of immune cell infiltration in lung tissue was evaluated by using the CIBERSORT algorithm. The differences in immune cell infiltration between groups were visualized with the “*ggplot2*” package, and Spearman correlation analysis was performed on all immune cells and diagnostic markers to determine the correlation between them (26, 27).

### Animal grouping and modeling

The study protocol met the National Institutes of Health Guide for the Care and Use of Laboratory Animals (NIH Publications No. 8023, revised 1978). Male Sprague–Dawley rats (180–220 g, specific pathogen-free grade) were purchased from Jinan Pengyue Experimental Animal Breeding Co., Ltd. [Certificate No. SCXK (Lu)2014-0007, Jinan, China]. The rats were placed in an environment with 12 h of lighting and 12 h of darkness per day and allowed to feed and drink freely. A single intratracheal instillation of bleomycin (BLM, Thermo Fisher Scientific Co., Ltd., LOT#2198541) was used to induce pulmonary fibrosis in the rats. After 7 days of adaptive breeding, in accordance with the documents, the rats were randomly divided into three groups (eight in each group): (1) the blank control group, (2) the BLM (2.5 mg/kg) group, and (3) the BLM (5 mg/kg) group (28). The rats were sacrificed 28 days later. Part of the lung tissues was placed in 4% paraformaldehyde, and the rest was frozen in liquid nitrogen and stored at −80°C for further examination.

### Morphological and histological analyses

The lung tissues were fixed by using 4% paraformaldehyde for 48 h, embedded in paraffin, and sliced to the thickness of 5 μm. The slices were stained with H&E and Masson trichrome to evaluate the pathological changes in lung tissues. At the same time, images were obtained with an optical microscope under 200 × magnification. The degrees of alveolitis and pulmonary fibrosis were scored in accordance with the Szapiel and Ashcroft scoring standards, respectively (29, 30).

### Measurement of non-esterified fatty acid content

The lung tissues were ground in cold physiological saline to obtain a 10% lung tissue homogenate. The homogenate was separated at 2500 rpm for 10 min at 4°C, and the supernatant was retained for the detection of free fatty acid levels according to the instructions of the corresponding kit (Nanjing Jiancheng Bioengineering Institute, Cat#A042-2-1).

### Quantitative real-time polymerase chain reaction

mRNA was extracted from the lung tissue with a universal RT-PCR Kit (Solarbio Science and Technology Co., Ltd., Shanghai, China) by following the manufacturer's instructions. Samples were treated with DNase and then purified by using an RNeasy kit (Qiagen, Hilden, Germany). Glyceraldehyde-3-phosphate dehydrogenase (*Gapdh*) was used as the internal reference. The PCR primer sequences included the following: *Muc5b*: forward primer: 5′-CCACCTACGAGGACTTCAACAT−3′; reverse primer: 5′-TTACCAGGACAGAGCCATTAGAC−3′, *Mmp-7*: forward primer: 5′-GGCATTCCAGAACTGTCACCTA−3′; reverse primer: 5′-CTTGCGAAGCCAATTATGATGT−3′, *Mmp-10*: forward primer: 5′-CCACTCAACCATGGATCTTGC−3′; reverse primer: 5′-ACAGTGTTCGAGTCCAGCTTCC−3′, *Mmp-13*: forward primer: 5′-GCCACCTTCTTCTTGTTGAGTTG−3′; reverse primer: 5′-GACTTCTTCAGGATTCCCGCA−3′, *Abca3*: forward primer: 5′-GGAGCTGGCTACCACATGACAC−3′; reverse primer: 5′-GGGAAGAATAAAGGACAACTCGG−3′, *Cyp24a1*: forward primer: 5′-CCTTCGCTCATCTCCCATTC−3′; reverse primer: 5′-ATTATCCAGCAGAGAGCCAGGTG−3′, *Gapdh*: forward primer: 5′-CTGGAGAAACCTGCCAAGTATG−3′; reverse primer: 5′-GGTGGAAGAATGGGAGTTGCT−3′.

### Statistical analysis

Data were shown as mean ± standard deviation. The differences between two groups were evaluated through the independent sample *t*-test and the nonparametric test. One-way ANOVA was used to compare the data among groups, and pairwise multiple comparisons between groups were made through the least significant difference test. *P* < 0.05 was considered statistically significant. Statistical analyses and figures were obtained by using IBM SPSS Statistics 23.0 (IBM SPSS Software, NY, USA).

## Results

### Identification of core FAMRGs in IPF

**Figure 1:**
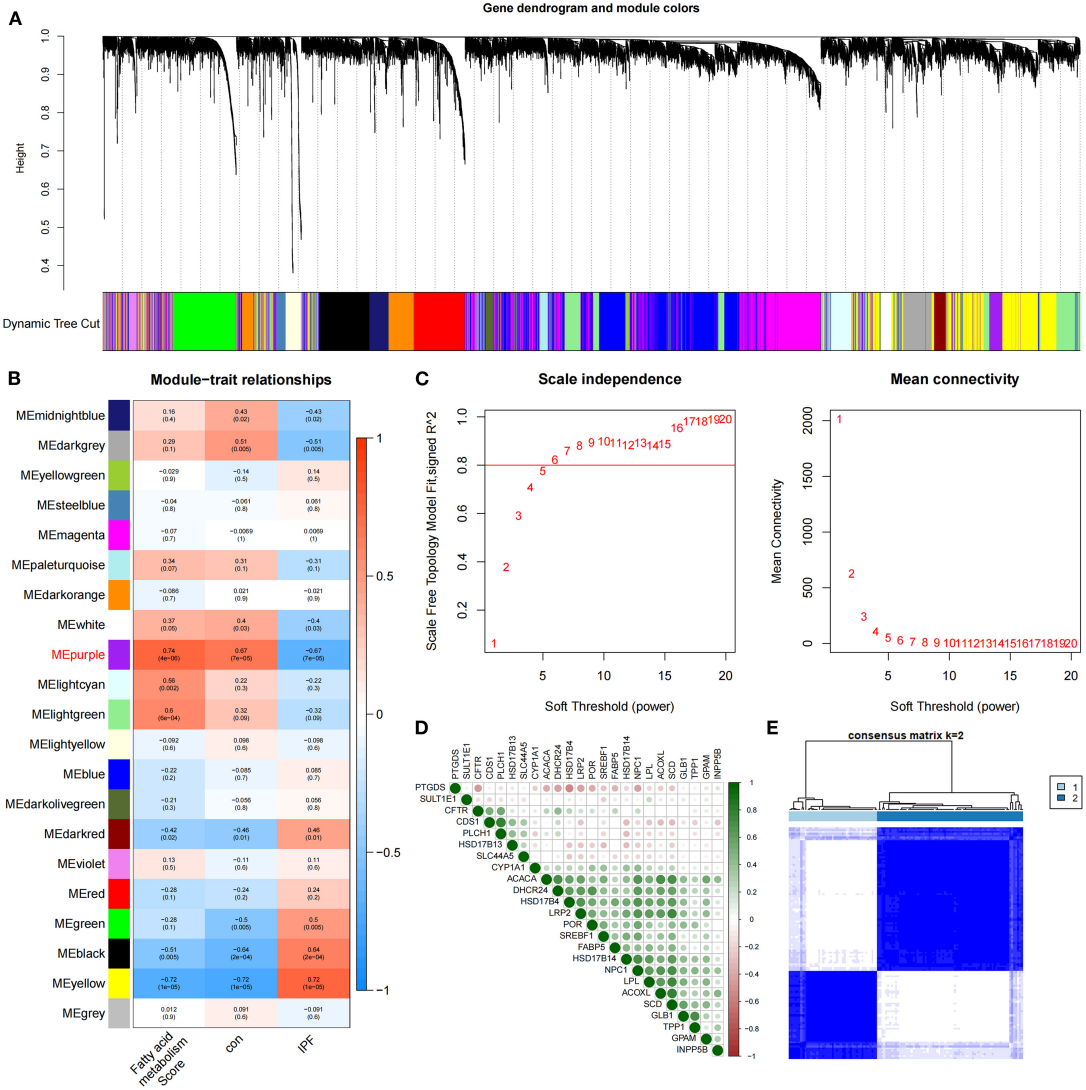
Identification of core FAMRGs in IPF. **(A)** WGCNA analysis was performed on the training set to obtain the cluster tree of co-expressed genes. **(B)** Construct module-trait relationships, with each module containing the corresponding correlation and *P*-value. **(C)** Soft threshold of scale-free network. **(D)** The interaction of 24 core FAMRGs. **(E)** Heatmap of the matrix of co-occurrence information for IPF samples.

The identification of 35 differentially expressed FAMRGs through the comparison of the gene expression levels in normal lung tissue with those in the lung tissue of patients with IPF (Supplementary Figure S1) indicated that fatty acid metabolism may be involved in the occurrence and development of IPF in the patients included in this dataset. Subsequently, we performed GSVA enrichment analysis in accordance with the expression levels of all FAMRGs, and the FMS of all samples was obtained. A scale-free network was constructed by combining the group information of FMS and samples, and the soft threshold was set to 5. WGCNA identified a total of 21 modules and marked them with unique colors. Analyzing the correlation among gene expression, groups, and FMS revealed that the MEpurple module (a total of 287 genes) was most associated with IPF and FMS (Figures 1A–C). The MEpurple module contained 24 FAMRGs with complex correlations (Figure 1D). Among these FAMRGs, *PTGDS* was negatively correlated with most genes, whereas *ACACA, DHCR24*, and *HSD17B14* were positively correlated with most genes.

### Core FAMRGs divide IPF into two subtypes

**Figure 2:**
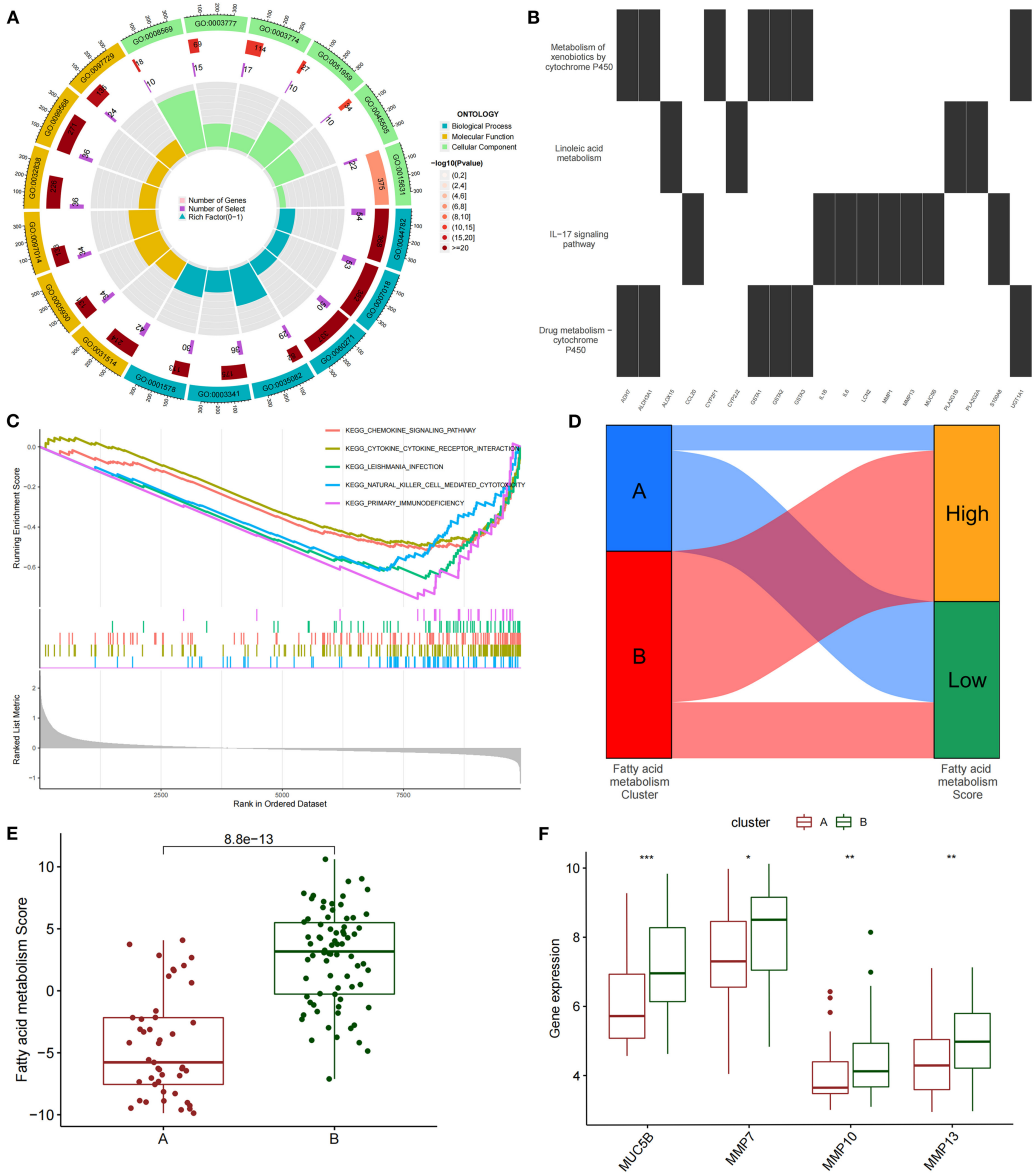
By unsupervised clustering of 24 FAMRGs, two different subtypes were identified in IPF. **(A)** GO analysis of differentially expressed genes between subtypes reveals related biological processes, molecular functions, and cellular components. **(B)** KEGG enrichment analysis of differentially expressed genes among subtypes. **(C)** GSEA analysis of key differential pathways among subtypes. **(D)** Distribution proportion of fatty acid metabolism in samples of different subtypes. **(E)** Differences in fatty acid metabolism between subtypes. **(F)** Differences in the expression levels of fibrosis-related biomarkers among subtypes. *P < 0.05, **P < 0.01, ***P < 0.001.

We conducted an unsupervised consensus cluster analysis on IPF samples on the basis of the expression of the 24 FAMRGs to study the role of FAMRGs in IPF. After the comprehensive evaluation of clinical significance and typing effect, *k* = 2 was selected as the optimal cluster number, and the two subtypes were named A and B (Figure 1E). PCA confirmed good discrimination between the two subtypes (Supplementary Figure S2). The differences in the expression of the 24 FAMRGs between subtypes A and B are shown in Supplementary Figure S3. We performed the functional enrichment analysis of DEGs between A and B subtypes to explore the differences in biological function between subtypes. GO enrichment analysis showed that cilium assembly, cilium movement, cilium-dependent cell mobility, and other cilium-related biological processes occupied the core position (Figure 2A). KEGG enrichment analysis revealed that the *IL-17* signaling pathway, linoleic acid metabolism, and other immune and fatty acid metabolism pathways were important signal transduction pathways of DEGs (Figure 2B). GSEA also verified the presence of significant differences in inflammation and immune-related processes (cytokine–cytokine receptor interaction and chemokine signaling pathway) between subtypes A and B and suggested that natural killer (NK) cells were core immune cells (Figure 2C). Interestingly, after scoring the fatty acid metabolism levels of the two molecular subtypes, most of the samples of subtype A were identified as low-FMS samples, whereas most of the samples of subtype B were identified as high-FMS samples (Figure 2D). This finding also statistically confirmed that the FMS of subtype B was significantly higher than that of subtype A (Figure 2E). The expression levels of diagnostic and prognostic biomarkers that were confirmed to be related to IPF in the two subtypes were compared to explore the association between the fatty acid metabolism level and IPF. The expression levels of *MUC5B, MMP-7, MMP-10, MMP-13*, and other fibrosis markers in subtype B were significantly up-regulated compared with those in subtype A, which was close to severe IPF (Figure 2F). These results suggested that core FAMRGs were involved in the progression of IPF and had a good classification function and that high fatty acid metabolism levels were associated with severe IPF.

### Construction and evaluation of a diagnostic nomogram model based on subtypes and the screening and verification of diagnostic markers

**Figure 3:**
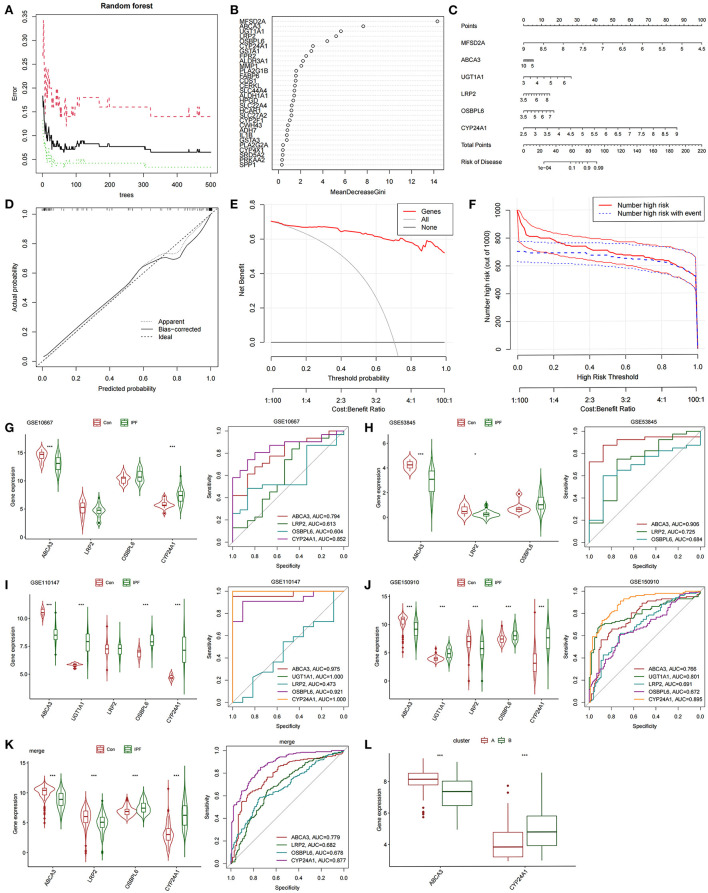
Construction and validation of diagnostic lirogram model. **(A)** Random forest tree constructed by cross validation. **(B)** Genes ranked in the top 30 by importance score. **(C)** Rograms were used to predict different fatty acid metabolism levels in IPF patients. **(D)** Calibration curves to assess the predictive power of the line-graph model. **(E)** DCA curve to evaluate the clinical value of the lipopograph model. **(F)** Evaluate the clinical impact curve of the lipopograph model based on DCA curve. **(G)** Expression levels and diagnostic efficacy of model key genes in dataset GSE10667. **(H)** Expression levels and diagnostic efficacy of model key genes in dataset GSE53845. **(I)** Expression levels and diagnostic efficacy of key genes in the model in dataset GSE110147. **(J**) Expression levels and diagnostic efficacy of model key genes in dataset GSE150910. **(K)** Expression levels and diagnostic efficacy of key genes in the model in the four combined data sets. **(L)** Expression levels of *ABCA3* and *CYP24A1* in subtypes A and B.

The differentially expressed FAMRGs between subtypes A and B were screened by constructing RF trees, and the genes with the top 30 importance scores were displayed (Figures 3A,B). Among these genes, *MFSD2A, ABCA3, UGT1A1, LRP2, OSBPL6*, and *CYP24A1* had high importance, and a diagnostic nomogram model was established on the basis of their expression (Figure 3C). The calibration curve showed that the error between the actual risk and the predicted risk was small. This result confirmed that the nomogram model has high accuracy in predicting fatty acid metabolism molecular subtypes (Figure 3D). DCA showed that the “Genes” curve of the related genes in the line graph model was higher than the gray curve, indicating that patients could benefit clinically within the high-risk threshold range from 0 to 1 (Figure 3E). The clinical effect curve was drawn on the basis of the DCA curve to evaluate the clinical effect of the nomogram model with increased intuitiveness. When the high-risk threshold ranged from 0.2 to 1, the curve of “Number High Risk” was close to that of “Number High Risk with Event,” indicating that the line graph model had good prediction ability (Figure 3F). The expression levels and diagnostic values of six genes were externally verified on the basis of the GSE10667, GSE53845, GSE110147, and GSE150910 datasets and their combined sets to screen out the key genes among the genes included in the nomogram model. The results showed that only the *ABCA3* and *CYP24A1* genes had constant differences between IPF samples and normal lung tissues and the AUC was higher than 0.75. The expression of *ABCA3* was down-regulated in subtype B and IPF samples, and the expression of *CYP24A1* was up-regulated in subtype B and IPF samples. These results confirmed the previous conclusion that subtype B was closer to severe IPF than subtype A and indicated that *ABCA3* and *CYP24A1* may mediate fatty acid metabolism and play an important role in the pathological evolution of IPF (Figures 3G–L).

### Fatty acid metabolism and expression characteristics of *Abca3* and *Cyp24a1* in rats with different degrees of pulmonary fibrosis

**Figure 4:**
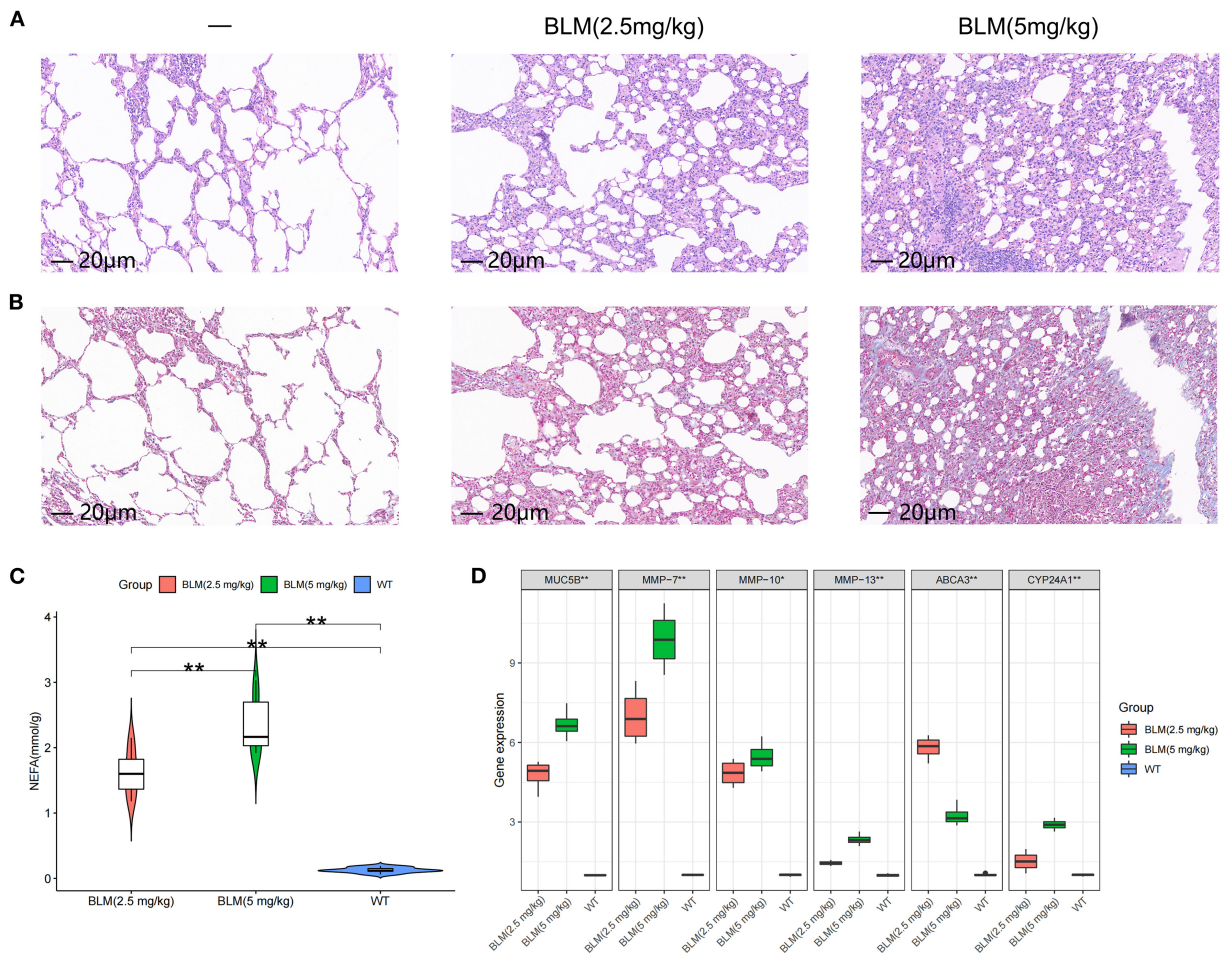
Expression levels of fibrosis markers and fatty acid metabolism in rats with different degrees of pulmonary fibrosis. **(A)** Photomicrographs of lung sections stained with H&E. **(B)** Photomicrographs of lung sections stained with Masson Trichrome staining. **(C)** Content of free fatty acids in lung tissues of rats in each group. **(D)** Gene expression levels of fibrosis markers and key diagnostic genes in lung tissues of rats in each group. *P < 0.05, **P < 0.01, ***P < 0.001.

We tested the level of fatty acid metabolism and the predictive value of *Abca3* and *Cyp24a1* in rat models with different degrees of pulmonary fibrosis to verify the accuracy of the prediction results. Two rat models with different degrees of pulmonary fibrosis were prepared through the intratracheal instillation of different concentrations of BLM. These models corresponded to patients with subtype A or subtype B IPF. HE and Masson staining revealed significant differences in the level of inflammation and fibrosis in lung tissue between the two models (Figures 4A,B), thus proving that the modeling was successful (Supplementary Figure S4). Further study revealed that 5 mg/kg BLM significantly increased the content of non-esterified fatty acids in lung tissue compared with 2.5 mg/kg BLM (Figure 4C). The expression levels of *Muc5b* mRNA, *Mmp-7* mRNA, *Mmp-10* mRNA, and *Mmp-13* mRNA in the BLM (5 mg/kg) group were higher than those in the BLM (2.5 mg/kg) group, and the expression of *Abca3* mRNA and *Cyp24a1* mRNA significantly differed between the two groups (Figure 4D). This difference verified the predictive value of *Abca3* and *Cyp24a1*.

### Differences in immune cell infiltration between subtypes and their correlation with *ABCA3* and *CYP24A1*

**Figure 5:**
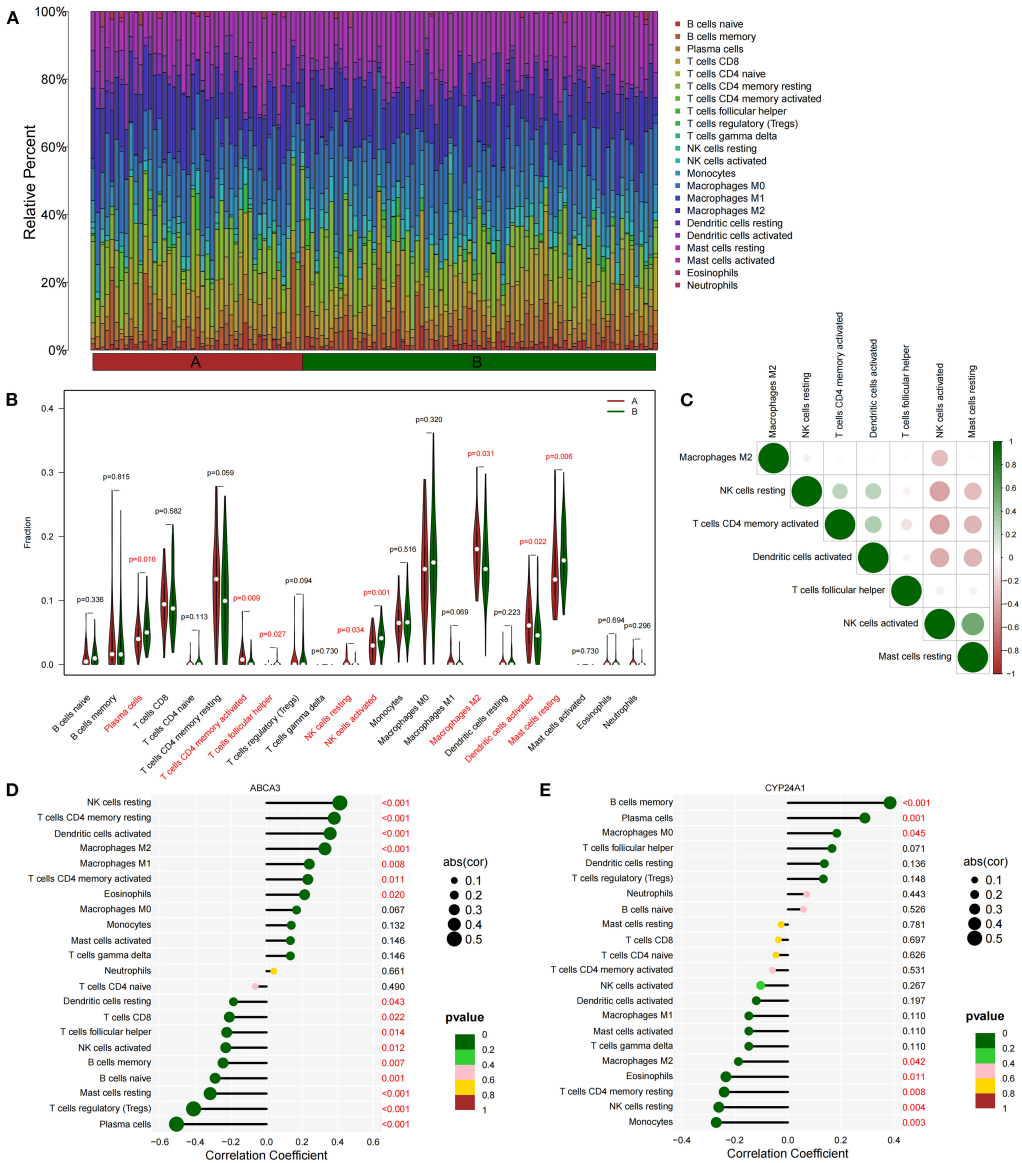
Immune cell infiltration between subtypes. **(A)** Immune microenvironment of subtypes A and B. **(B)** Differences in immune cell infiltration among subtypes. **(C)** Correlation heat maps of immune cells with differences. **(D)** Correlation between *ABCA3* gene expression and immune cell infiltration. **(E)** Correlation between *CYP24A1* gene expression level and immune cell infiltration.

We quantified the level of immune cell infiltration (Figure 5A) to evaluate the immune landscapes of subtypes A and B. Our results showed that plasma cells, CD4^+^ memory-activated T cells, follicular helper T cells, resting and activated NK cells, M2 macrophages, activated dendritic cells, and resting mast cells had significant differences between the A and B subtypes. These differences indicated that under the influence of fatty acid metabolic processes, abundant immune cell heterogeneity existed within the lung tissue (Figure 5B). At the same time, a certain correlation was found between immune cells with different degrees of infiltration, and activated NK cells were negatively correlated with most immune cells (Figure 5C). Spearman correlation analysis demonstrated that *ABCA3* was more commonly correlated with various immune cells than *CYP24A1*. *ABCA3* was positively correlated with the infiltration of eosinophils, CD4^+^ memory activated and resting T cells, M1 and M2 macrophages, activated T cells, and resting NK cells and negatively correlated with the infiltration of plasma cells, regulatory T cells, resting mast cells, naïve and memory B cells, activated NK cells, follicular helper and CD8^+^ T cells, and resting dendritic cells (Figures 5D,E).

## Discussion

Fatty acid metabolism has been extensively studied for a long time due to its close relationship with IPF. Studies related to fatty acid metabolism encompass those that measure the differences in plasma fatty acid content and species between patients with IPF and healthy people to explore their possibility as biomarkers to those that confirm that FAMRGs play a core role in the fibrosis of IPF at the single-cell level (31, 32). In view of this situation, in this study, we first identified 24 central genes that may be involved in the pathological processes related to fatty acid metabolism in IPF and confirmed the relationship between some of these genes and IPF. For example, *SREBF1* is a transcription factor related to lipid metabolism, which is closely related to the activation of fibroblasts. Activating the *LXR/SREBP-1c* axis can inhibit *SREBF1*-dependent activation and IPF progression in fibroblasts (33). *SCD* encodes enzymes involved in fatty acid biosynthesis and is mainly involved in the synthesis of oleic acid. Only two of its isoforms, *SCD1* and *SCD5*, have been identified in humans. *SCD1* concentrations are down-regulated in lung tissue from patients with IPF, and *SCD1* inhibition leads to endoplasmic reticulum stress and promotes pulmonary fibrosis (34). Meanwhile, the classification of patients with IPF based on these core FAMRGs also suggests that the linoleic acid metabolism pathway is a differential signaling pathway between subtypes with different fatty acid metabolism levels. Non-targeted metabolomics confirmed that linoleic acid is one of the most significantly different components in the plasma fatty acid composition of patients with IPF relative to those in healthy controls with a difference of approximately 2–3 times (35). Supplementation with *Pistacia lentiscus* oil, which contains linoleic acid as the main component, could reduce the levels of inflammatory cell infiltration and TGF-β in the lung tissue of rats with pulmonary fibrosis (36). Notably, a strong relationship exists between the level of fatty acid metabolism in subtypes and biomarkers that imply IPF progression and poor prognosis. Among the biomarkers included in this study, *MUC5B* is significantly up-regulated in the lung tissues of patients with IPF. The up-regulation of *MUC5B* leads to excessive mucus secretion and damage to the epithelial ciliary movement, thus inducing and enhancing chronic inflammation and injury (37, 38). Matrix metalloproteinases (*MMPs*) are another type of biomarker included in this study. The *MMP* family has numerous members, and its expression is generally up-regulated in IPF. *MMPs* are a key molecule involved in lung extracellular matrix remodeling in patients with IPF. Among *MMPs, MMP-13* is a key *MMP* that is up-regulated in human IPF and is mainly involved in regulating epithelial-mesenchymal transition and collagen deposition (39). *MMP-7* is expressed by airway epithelial cells and macrophages in the lung. It is related to pulmonary diffusion function and survival rate in patients with IPF. Moreover, plasma *MMP-7* levels have been identified as a biomarker for IPF (40, 41). Increased serum *MMP-10* levels are associated with clinical deterioration within 6 months and overall survival (42). Our study showed that high fatty acid metabolism levels are positively correlated with the expression of the above biomarkers. Previous studies have revealed the pharmacological mechanisms of some small-molecule compounds in the treatment of IPF by targeting the fatty acid metabolism pathway, suggesting that fatty acid metabolism-related targets are potentially valuable in the development of pharmaceuticals for IPF (43).

The results of our single-cell RNA sequencing study suggested that fatty acid metabolism and immune processes are jointly involved in the early progression of IPF (44). In this study, we found that NK cells were significantly activated in the lung tissues of patients with IPF, as well as high levels of fatty acid metabolism. Our GSEA also confirmed that NK cell-induced cytotoxic effects were the most significantly differentially regulated immune processes between different subtypes. The perforin-mediated pathway is the main pathway of the cytotoxic effects induced by activated NK cells, and perforin-mediated apoptosis promotes the development of pulmonary fibrosis by triggering lung tissue inflammation (45). In addition, the *CX3CL1/CX3CR1* axis regulates NK cell activation and infiltration (46). *PXN* is considered as a risk gene in IPF and is overexpressed in activated NK cells. The inhibition of the *FAK/PXN* signaling pathway could reduce proinflammatory cytokine secretion in activated NK cells to exert protective effects on fibrotic lung tissues (47). NK cells are also closely associated with key profibrotic processes in IPF, such as cellular hypoxia. A clinical study found that activated NK cells, key hypoxia genes, and risk scores were positively correlated, and the elevated proportion of activated NK cells in the bronchoalveolar lavage fluid (BALF) of patients with IPF implied long hospitalization days. These results all indicated that high NK cell infiltration is a risk factor for poor prognosis in IPF (48). Another clinical study also confirmed that the proportion of NK cells in the BALF of patients with IPF was significantly higher than that in the BALF of patients with other interstitial lung diseases and observed a significant difference in survival rates with the cutoff value of 4% (49). These previous findings, which are consistent with our conclusions, provide additional levels of evidence for the correlation between fatty acid metabolism and the immune microenvironment in IPF. Interestingly, both FAMRGs used to distinguish fatty acid metabolic subtypes showed a close correlation with NK cells. *ABCA3* is a phospholipid transporter associated with pulmonary surfactant homeostasis, and mutations in the *ABCA3* gene cause pulmonary fibrosis in children and adults (50, 51). *CYP24A1* is an enzyme that regulates vitamin D metabolism through the activation of a negative feedback loop, while vitamin D3 has been shown to down-regulate the expression of proinflammatory and profibrotic markers, such as *CCL2, TLR3*, fibronectin, and type I collagen. These two predictive markers were confirmed to be stable in the validation group and animal experiments, and corresponding clinical studies have been conducted in the field of organ fibrosis, proving that their detection is simple and reliable, thus providing additional possibilities for the use of new targeted approaches in IPF treatment and management. However, whether they mediate NK cell activation to regulate the occurrence and development of IPF still requires further experimental investigation.

This study has several limitations. First, it was conducted on the basis of the GEO database. Although we collected multiple datasets, the sample numbers were relatively small and our conclusions may still be biased. Second, our animal experiments only studied the correlation between total non-esterified fatty acids content and predicted markers and failed to identify specific fatty acid types accurately. Third, most datasets were not accompanied by the important clinical information of the patients, such as pulmonary function parameters, St. George's respiratory questionnaire scores, and whether or not anti-fibrotic drugs were taken. Therefore, the correlation of predictive markers with clinical parameters and patient prognosis needs to be further investigated.

## Conclusion

In this study, we identified 24 central genes that are closely related to fatty acid metabolism in IPF. These genes can divide IPF into two subtypes. The constructed line chart model has a good ability to distinguish between subtypes. *ABCA3* and *CYP24A1* were verified as key diagnostic markers between subtypes by using external datasets and animal experiments. The level of immune cell infiltration among subtypes is strongly heterogeneous. NK cells are the key immune effector cells, and *ABCA3* and *CYP24A1* are closely related to many kinds of immune cells. To our knowledge, this is the first study to explore the relationship between fatty acid metabolism and its related genes and the progression of pulmonary fibrosis in combination with the immune microenvironment of lung tissue in IPF. This study initially established the relationship between fatty acid metabolism and its related genes in IPF with pulmonary fibrosis progression. However, further studies are needed to test the clinical value of our results.

## Data availability statement

Publicly available datasets were analyzed in this study. This data can be found at: (https://www.ncbi.nlm.nih.gov/geo/), datasets GSE32537, GSE53845, GSE10667, GSE110147, and GSE150910.

## Ethics statement

The animal study was reviewed and approved by Experimental Animal Ethics Committee of the Affiliated Hospital of Shandong University of Traditional Chinese Medicine.

## Author contributions

FY designed and conducted the whole research. ZM, WL, and BW carried out animal experiments and molecular biological analysis. JK and YZ applied for the GEO dataset analysis of IPF. FY, QW, YL, and GD completed the data analysis and drafted the manuscript. XZ and JW revised and finalized the manuscript. All authors contributed to the article and approved the submitted version.

## Funding

This work was supported by the National Key R&D Program of China (2020YFC2003100 and 2020YFC2003101), Innovation Team and Talents Cultivation Program of National Administration of Traditional Chinese Medicine (No. ZYYCXTD-C-202001).

## Conflict of interest

The authors declare that the research was conducted in the absence of any commercial or financial relationships that could be construed as a potential conflict of interest.

## Publisher's note

All claims expressed in this article are solely those of the authors and do not necessarily represent those of their affiliated organizations, or those of the publisher, the editors and the reviewers. Any product that may be evaluated in this article, or claim that may be made by its manufacturer, is not guaranteed or endorsed by the publisher.
