# Supplementary material for: Identification and immune characteristics of molecular subtypes related to fatty acid metabolism in idiopathic pulmonary fibrosis
Source: Front Nutr. 2022 Sep 23;9:992331. doi: 10.3389/fnut.2022.992331 (PMC9537386; doi:10.3389/fnut.2022.992331)
Supplement: Supplementary file 1 [file Data_Sheet_1.docx]

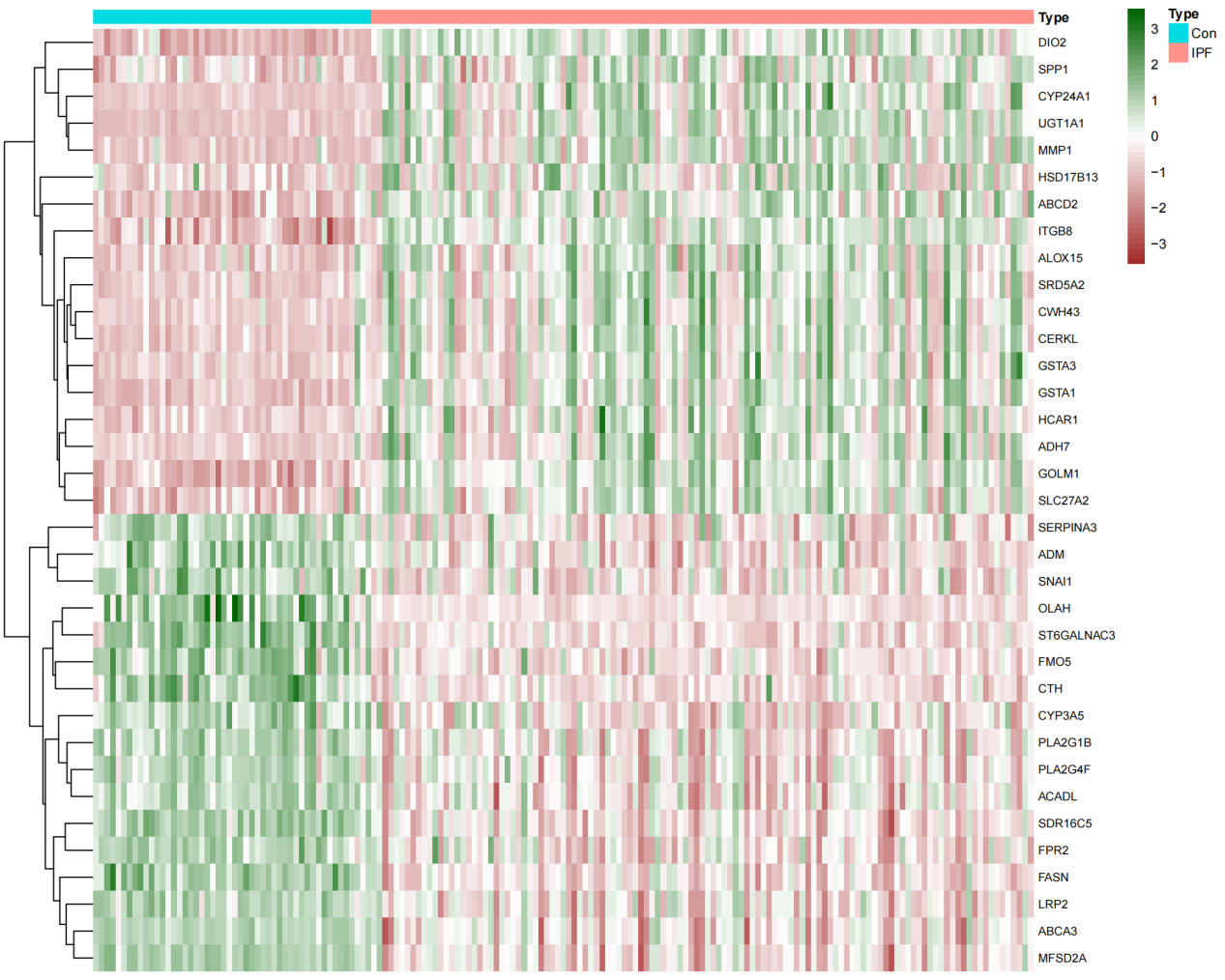


Figure S1 Differentially expressed FMARGs between normal lung tissue and lung tissue of IPF patients.


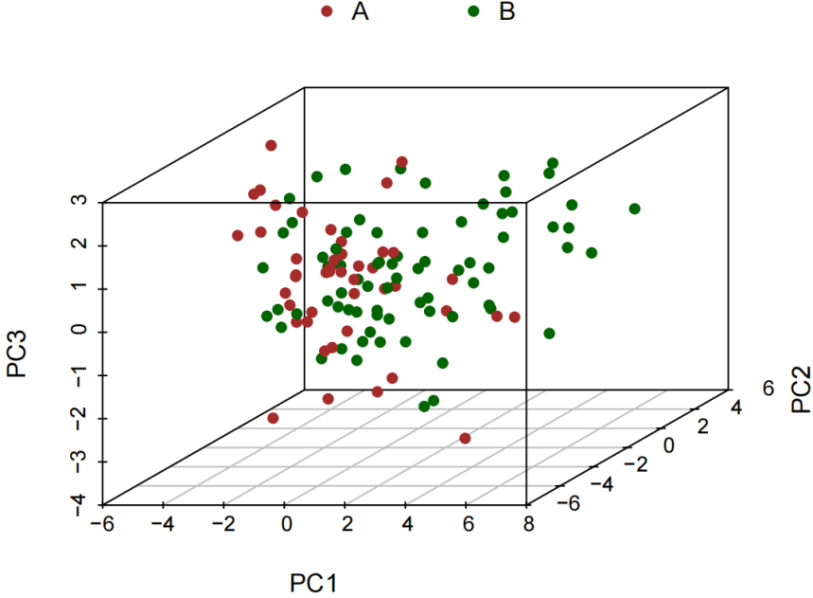


Figure S2 The PCA scatter diagram shows the similarity between the two subtypes.


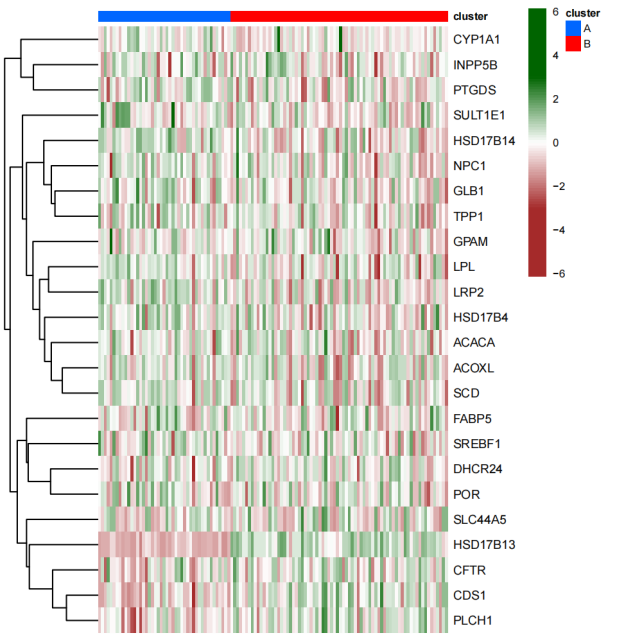


Figure S3 Expression difference of 24 FAMRGs between subtype A and subtype B.


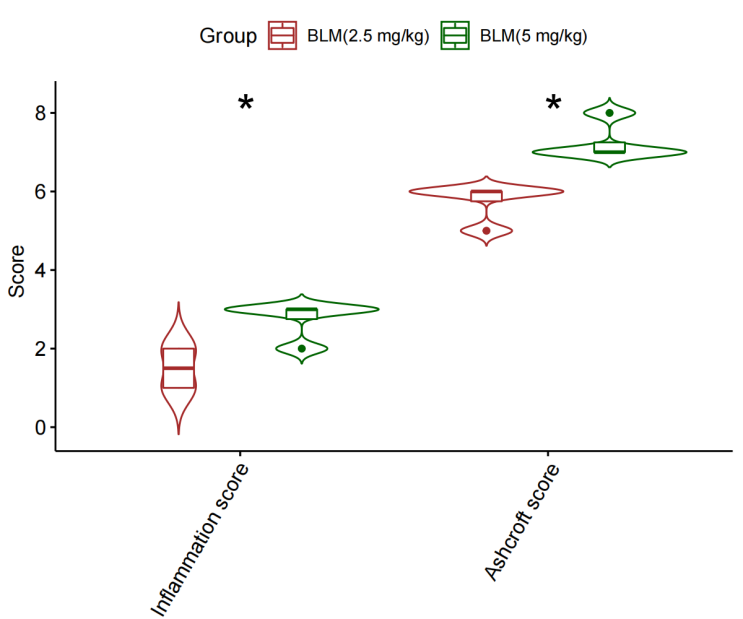


Figure S4 The difference of inflammation score and fibrosis score in lung tissue of rats induced by different doses of BLM. **P* < 0.05.
